# Supplementary material for: Small Extracellular Vesicles from Hypoxic Triple-Negative Breast Cancer Cells Induce Oxygen-Dependent Cell Invasion
Source: Int J Mol Sci. 2022 Oct 21;23(20):12646. doi: 10.3390/ijms232012646 (PMC9604480; doi:10.3390/ijms232012646)
Supplement: Supplementary file 1 [file ijms-23-12646-s001.zip › Supplementary Figure Legends.pdf]

Supplementary Figure S1. Additional methodological details (a) Integrin immunophenotyping gating strategy for flow cytometry analysis; (b) Parameters for quantification of cell morphology: (1) Cell circularity, (2) Aspect Ratio and (3) Perimeter/Area; (c) Apoptosis assay controls and gating strategy, including unstained controls incubated in normoxia and hypoxia, single stained controls for annexin-V and 7AAD, and double stained controls for live and dead cells. Values represent the percentage of cells in each quadrant.

Supplementary Figure S2. Cell invasion in acute hypoxia (a) Invasion index (%) for 6h in Matrigel (mean ± SEM) (b) Comparison of degraded area after 48 h incubation in normoxia (20% O_2) or hypoxia (1% O_2) of negative control (FBS-), positive control (FBS+) and treated cells (SEVh); data shown as median ± interquartile range (c) Representative images of 24h gelatin degradation assay in light microscopy (LM), and fluorescent-labelled nuclei (DAPI, blue), tubulin (AlexaFluor647, pink) and gelatin (0.2 mg/ml, fluorescein, green); overlayed images below. Degradation spots in gelatin coating (black) are signaled with white arrows. Scale bars: 20 µm. Proteolytic activity from gelatin degradation assay supernatant of (d) MMP-2 (mean ± SD) and (e) MMP-9 (mean ± SD), with zymograms displayed over each graph. *$p<0.05$

Supplementary Figure S3. Migratory morphology is induced by SEVh in Matrigel. Additional parameters for assessing cell morphology (n=300). (a-c) Aspect ratio (AR) of cells after 24h and 48h for cells adhered to (a) an uncoated substrate, (b) gelatin coating (0.2 mg/ml) or (c) Matrigel coating (1:1, v/v); data displayed as median ± interquartile range. (d-f) Perimeter per area ratio obtained from cells incubated for 24h and 48h for cells adhered to (d) an uncoated substrate, (e) gelatin coating (0.2 mg/ml) or (f) Matrigel coating (1:1, v/v); data shown as median ± interquartile range. *$p<0.05$
