# Supplementary material for: Small Extracellular Vesicles from Hypoxic Triple-Negative Breast Cancer Cells Induce Oxygen-Dependent Cell Invasion
Source: Int J Mol Sci. 2022 Oct 21;23(20):12646. doi: 10.3390/ijms232012646 (PMC9604480; doi:10.3390/ijms232012646)
Supplement: Supplementary file 1 [file ijms-23-12646-s001.zip › Table S1.Differential proteomic profile of SEVh.pdf]

**Supplementary Table S1.** Differential proteomic profile of SEVh ($p<0.01$)

| **Protein Code** | **Protein Name** | **t.stat** | ***p* value** | **- log10(*p*)** | **FDR*** |
|---|---|---|---|---|---|
| ISG15 | Ubiquitin-like protein ISG15 | 196.650 | 4.01E-09 | 8.397 | 9.59E-06 |
| PR40A | Pre-mRNA-processing factor 40 homolog A | 80.288 | 1.44E-07 | 6.841 | 1.72E-04 |
| MTOR | Serine/threonine-protein kinase mTOR | 60.318 | 4.52E-07 | 6.344 | 3.60E-04 |
| IMA5 | Importin subunit alpha-5 | 48.570 | 1.08E-06 | 5.969 | 5.40E-04 |
| RL36 | 60S ribosomal protein L36 | 47.965 | 1.13E-06 | 5.947 | 5.40E-04 |
| HNRL2 | Heterogeneous nuclear ribonucleoprotein U-like protein 2 | 40.705 | 2.18E-06 | 5.662 | 8.42E-04 |
| PKN2 | Serine/threonine-protein kinase N2 | 38.313 | 2.77E-06 | 5.557 | 8.42E-04 |
| CHERP | Calcium homeostasis endoplasmic reticulum protein | 35.985 | 3.56E-06 | 5.449 | 8.42E-04 |
| RO60 | RNA-binding protein RO60 | 35.466 | 3.77E-06 | 5.423 | 8.42E-04 |
| MRCKB | Serine/threonine-protein kinase MRCK beta | 34.954 | 4.00E-06 | 5.398 | 8.42E-04 |
| ENOG | Gamma-enolase | 34.607 | 4.16E-06 | 5.381 | 8.42E-04 |
| SNRPA | U1 small nuclear ribonucleoprotein A | 34.466 | 4.23E-06 | 5.374 | 8.42E-04 |
| MSH2 | DNA mismatch repair protein Msh2 | 33.412 | 4.79E-06 | 5.320 | 8.80E-04 |
| TEBP | Prostaglandin E synthase 3 | 30.983 | 6.47E-06 | 5.189 | 1.05E-03 |
| RPTOR | Regulatory-associated protein of mTOR | 30.832 | 6.59E-06 | 5.181 | 1.05E-03 |
| DEFI6 | Differentially expressed in FDCP 6 homolog | 29.611 | 7.75E-06 | 5.111 | 1.12E-03 |
| SMAD3; SMAD5; SMAD1; SMAD2; SMAD9 | Mothers against decapentaplegic homolog 3; Mothers against decapentaplegic homolog 5; Mothers against decapentaplegic homolog 1; Mothers against decapentaplegic homolog 2; Mothers against decapentaplegic homolog 9 | 29.375 | 8.00E-06 | 5.097 | 1.12E-03 |
| MAP1B | Microtubule-associated protein 1B | 27.882 | 9.84E-06 | 5.007 | 1.15E-03 |
| DKC1 | H/ACA ribonucleoprotein complex subunit DKC1 | 27.850 | 9.89E-06 | 5.005 | 1.15E-03 |
| DCTN2 | Dynactin subunit 2 | 27.674 | 1.01E-05 | 4.994 | 1.15E-03 |
| TRA2B | Transformer-2 protein homolog beta | 27.669 | 1.01E-05 | 4.994 | 1.15E-03 |
| EIF3H | Eukaryotic translation initiation factor 3 subunit H | 25.945 | 1.31E-05 | 4.882 | 1.41E-03 |
| MTND | 1.2-dihydroxy-3-keto-5-methylthiopentene dioxygenase | 25.713 | 1.36E-05 | 4.867 | 1.41E-03 |
| AQR | RNA helicase aquarius | 24.715 | 1.59E-05 | 4.798 | 1.58E-03 |
| CMBL | Carboxymethylenebutenolidase homolog | 23.785 | 1.85E-05 | 4.732 | 1.67E-03 |
| OXSR1 | Serine/threonine-protein kinase OSR1 | 23.503 | 1.94E-05 | 4.712 | 1.67E-03 |
| CCZ1; CCZ1B | Vacuolar fusion protein CCZ1 homolog; Vacuolar fusion protein CCZ1 homolog B | 23.227 | 2.04E-05 | 4.691 | 1.67E-03 |
| MET | Hepatocyte growth factor receptor | 23.003 | 2.12E-05 | 4.674 | 1.67E-03 |

| | | | | | |
|---|---|---|---|---|---|
| CAND1 | Cullin-associated NEDD8-dissociated protein 1 | 22.901 | 2.15E-05 | 4.667 | 1.67E-03 |
| RGPD4; RGPD3; RGPD1; RGPD2; RGPD5; RGPD8 | RanBP2-like and GRIP domain-containing protein 4; RanBP2-like and GRIP domain-containing protein 3; RanBP2-like and GRIP domain-containing protein 1; RanBP2-like and GRIP domain-containing protein 2; RanBP2-like and GRIP domain-containing protein 5; RanBP2-like and GRIP domain-containing protein 8 | 22.863 | 2.17E-05 | 4.664 | 1.67E-03 |
| CSTF2 | Cleavage stimulation factor subunit 2 | 22.852 | 2.17E-05 | 4.663 | 1.67E-03 |
| IPO5 | Importin-5 | 22.673 | 2.24E-05 | 4.650 | 1.67E-03 |
| PA1B3 | Platelet-activating factor acetylhydrolase IB subunit alpha1 | 22.051 | 2.50E-05 | 4.602 | 1.78E-03 |
| MAP4 | Microtubule-associated protein 4 | 21.890 | 2.58E-05 | 4.589 | 1.78E-03 |
| YTHD3; YTHD1 | YTH domain-containing family protein 3; YTH domain-containing family protein 1 | 21.827 | 2.61E-05 | 4.584 | 1.78E-03 |
| KPCD | Protein kinase C delta type | 21.357 | 2.84E-05 | 4.546 | 1.85E-03 |
| GEPH | Gephyrin | 21.326 | 2.86E-05 | 4.544 | 1.85E-03 |
| CSN7A | COP9 signalosome complex subunit 7a | 20.934 | 3.08E-05 | 4.512 | 1.94E-03 |
| AR6P4 | ADP-ribosylation factor-like protein 6-interacting protein 4 | 20.082 | 3.63E-05 | 4.440 | 2.19E-03 |
| AIMP2 | Aminoacyl tRNA synthase complex-interacting multifunctional protein 2 | 20.036 | 3.66E-05 | 4.436 | 2.19E-03 |
| CO5A1 | Collagen alpha-1(V) chain | 19.801 | 3.84E-05 | 4.416 | 2.24E-03 |
| RS2 | 40S ribosomal protein S2 | 19.162 | 4.37E-05 | 4.359 | 2.49E-03 |
| ANFY1 | Rabankyrin-5 | 18.862 | 4.65E-05 | 4.332 | 2.54E-03 |
| SCFD1 | Sec1 family domain-containing protein 1 | 18.843 | 4.67E-05 | 4.331 | 2.54E-03 |
| CHD1 | Chromodomain-helicase-DNA-binding protein 1 | 18.682 | 4.83E-05 | 4.316 | 2.54E-03 |
| MET25 | Probable methyltransferase-like protein 25 | 18.622 | 4.89E-05 | 4.310 | 2.54E-03 |
| EI2BD | Translation initiation factor eIF-2B subunit delta | 18.212 | 5.35E-05 | 4.272 | 2.72E-03 |
| GALT2 | Polypeptide N-acetylgalactosaminyltransferase 2 | 18.063 | 5.52E-05 | 4.258 | 2.75E-03 |
| CUTA | Protein CutA | 17.857 | 5.78E-05 | 4.238 | 2.82E-03 |
| RLA2 | 60S acidic ribosomal protein P2 | 17.691 | 6.00E-05 | 4.222 | 2.87E-03 |
| RIR2 | Ribonucleoside-diphosphate reductase subunit M2 | 17.556 | 6.18E-05 | 4.209 | 2.90E-03 |
| AP2S1 | AP-2 complex subunit sigma | 17.469 | 6.31E-05 | 4.200 | 2.90E-03 |
| KITH | Thymidine kinase. cytosolic | 17.357 | 6.47E-05 | 4.189 | 2.92E-03 |
| GFPT2 | Glutamine--fructose-6-phosphate aminotransferase [isomerizing] 2 | 17.203 | 6.70E-05 | 4.174 | 2.96E-03 |
| MYLK | Myosin light chain kinase. smooth muscle | 17.074 | 6.90E-05 | 4.161 | 3.00E-03 |
| TNS2; TENS3; TENS1 | Tensin-2; Tensin-3; Tensin-1 | 16.846 | 7.28E-05 | 4.138 | 3.11E-03 |
| VPS36 | Vacuolar protein-sorting-associated protein 36 | 16.482 | 7.94E-05 | 4.100 | 3.27E-03 |

| | | | | | |
|---|---|---|---|---|---|
| PAF1 | RNA polymerase II-associated factor 1 homolog | 16.479 | 7.94E-05 | 4.100 | 3.27E-03 |
| PALLD | Palladin | 16.321 | 8.25E-05 | 4.084 | 3.34E-03 |
| IF4G2 | Eukaryotic translation initiation factor 4 gamma 2 | 16.113 | 8.68E-05 | 4.062 | 3.46E-03 |
| FPPS | Farnesyl pyrophosphate synthase | 15.836 | 9.29E-05 | 4.032 | 3.52E-03 |
| ARL1 | ADP-ribosylation factor-like protein 1 | 15.821 | 9.33E-05 | 4.030 | 3.52E-03 |
| SYMC | Methionine--tRNA ligase. cytoplasmic | 15.788 | 9.40E-05 | 4.027 | 3.52E-03 |
| SATT | Neutral amino acid transporter A | 15.774 | 9.44E-05 | 4.025 | 3.52E-03 |
| OGFR | Opioid growth factor receptor | 15.662 | 9.71E-05 | 4.013 | 3.57E-03 |
| RAB1B | Ras-related protein Rab-1B | 15.602 | 9.85E-05 | 4.006 | 3.57E-03 |
| CSN5 | COP9 signalosome complex subunit 5 | 15.515 | 1.01E-04 | 3.997 | 3.59E-03 |
| RRAGA; RRAGB | Ras-related GTP-binding protein A; Ras-related GTP-binding protein B | 15.436 | 1.03E-04 | 3.988 | 3.61E-03 |
| FXR2 | Fragile X mental retardation syndrome-related protein 2 | 15.097 | 1.12E-04 | 3.950 | 3.89E-03 |
| EXOS3 | Exosome complex component RRP40 | 15.027 | 1.14E-04 | 3.942 | 3.90E-03 |
| RB27A | Ras-related protein Rab-27A | 14.831 | 1.20E-04 | 3.920 | 4.05E-03 |
| CRLD1 | Cysteine-rich secretory protein LCCL domain-containing 1 | 14.651 | 1.26E-04 | 3.899 | 4.09E-03 |
| MCM3 | DNA replication licensing factor MCM3 | 14.641 | 1.27E-04 | 3.898 | 4.09E-03 |
| ZNT7 | Zinc transporter 7 | 14.639 | 1.27E-04 | 3.897 | 4.09E-03 |
| LANC1 | Glutathione S-transferase LANCL1 | 14.513 | 1.31E-04 | 3.883 | 4.14E-03 |
| AP1G1 | AP-1 complex subunit gamma-1 | 14.497 | 1.32E-04 | 3.881 | 4.14E-03 |
| GAPD1 | GTPase-activating protein and VPS9 domain-containing protein 1 | 14.033 | 1.50E-04 | 3.825 | 4.64E-03 |
| ARHGI | Rho guanine nucleotide exchange factor 18 | 13.628 | 1.68E-04 | 3.775 | 5.08E-03 |
| IFRD2 | Interferon-related developmental regulator 2 | 13.626 | 1.68E-04 | 3.775 | 5.08E-03 |
| IF4G1 | Eukaryotic translation initiation factor 4 gamma 1 | 13.450 | 1.77E-04 | 3.753 | 5.28E-03 |
| FERM1 | Fermitin family homolog 1 | 13.217 | 1.89E-04 | 3.723 | 5.59E-03 |
| ARGL1 | Arginine and glutamate-rich protein 1 | 13.099 | 1.96E-04 | 3.708 | 5.72E-03 |
| KCC2D | Calcium/calmodulin-dependent protein kinase type II subunit delta | 13.026 | 2.00E-04 | 3.698 | 5.74E-03 |
| HPRT | Hypoxanthine-guanine phosphoribosyltransferase | 13.005 | 2.02E-04 | 3.695 | 5.74E-03 |
| RBM25 | RNA-binding protein 25 | 12.929 | 2.06E-04 | 3.685 | 5.76E-03 |
| SEC13 | Protein SEC13 homolog | 12.896 | 2.08E-04 | 3.681 | 5.76E-03 |
| SART3 | Squamous cell carcinoma antigen recognized by T-cells 3 | 12.877 | 2.10E-04 | 3.678 | 5.76E-03 |
| CK098 | Uncharacterized protein C11orf98 | 12.827 | 2.13E-04 | 3.672 | 5.78E-03 |
| AP1B1 | AP-1 complex subunit beta-1 | 12.759 | 2.17E-04 | 3.663 | 5.83E-03 |

| | | | | | |
|---|---|---|---|---|---|
| PR38B | Pre-mRNA-splicing factor 38B | 12.707 | 2.21E-04 | 3.656 | 5.83E-03 |
| PHF5A | PHD finger-like domain-containing protein 5A | 12.690 | 2.22E-04 | 3.654 | 5.83E-03 |
| CAPR1 | Caprin-1 | 12.606 | 2.28E-04 | 3.642 | 5.88E-03 |
| NOP16 | Nucleolar protein 16 | 12.593 | 2.29E-04 | 3.640 | 5.88E-03 |
| TBC15 | TBC1 domain family member 15 | 12.388 | 2.44E-04 | 3.613 | 6.21E-03 |
| PGTB2 | Geranylgeranyl transferase type-2 subunit beta | 12.319 | 2.49E-04 | 3.603 | 6.28E-03 |
| PPT1 | Palmitoyl-protein thioesterase 1 | 12.096 | 2.68E-04 | 3.572 | 6.53E-03 |
| IF2B2 | Insulin-like growth factor 2 mRNA-binding protein 2 | 12.091 | 2.68E-04 | 3.571 | 6.53E-03 |
| GCP60 | Golgi resident protein GCP60 | 12.051 | 2.72E-04 | 3.566 | 6.53E-03 |
| SHSA4 | Protein shisa-4 | 12.038 | 2.73E-04 | 3.564 | 6.53E-03 |
| ARF6 | ADP-ribosylation factor 6 | 12.037 | 2.73E-04 | 3.564 | 6.53E-03 |
| TCTP | Translationally-controlled tumor protein | 11.969 | 2.79E-04 | 3.554 | 6.59E-03 |
| RFC2 | Replication factor C subunit 2 | 11.934 | 2.82E-04 | 3.549 | 6.59E-03 |
| SHC1; SHC2 | SHC-transforming protein 1; SHC-transforming protein 2 | 11.898 | 2.86E-04 | 3.544 | 6.59E-03 |
| CPSF1 | Cleavage and polyadenylation specificity factor subunit 1 | 11.890 | 2.87E-04 | 3.543 | 6.59E-03 |
| UBP2L | Ubiquitin-associated protein 2-like | 11.724 | 3.03E-04 | 3.519 | 6.89E-03 |
| PDLI1 | PDZ and LIM domain protein 1 | 11.656 | 3.10E-04 | 3.509 | 6.98E-03 |
| WDR11 | WD repeat-containing protein 11 | 11.589 | 3.17E-04 | 3.499 | 7.07E-03 |
| AP2A2 | AP-2 complex subunit alpha-2 | 11.453 | 3.32E-04 | 3.479 | 7.30E-03 |
| MARE1 | Microtubule-associated protein RP/EB family member 1 | 11.443 | 3.33E-04 | 3.478 | 7.30E-03 |
| SF3B2 | Splicing factor 3B subunit 2 | 11.380 | 3.40E-04 | 3.469 | 7.39E-03 |
| ARL3 | ADP-ribosylation factor-like protein 3 | 11.355 | 3.43E-04 | 3.465 | 7.39E-03 |
| CDC16 | Cell division cycle protein 16 homolog | 11.224 | 3.59E-04 | 3.445 | 7.66E-03 |
| LARP1 | La-related protein 1 | 11.179 | 3.65E-04 | 3.438 | 7.71E-03 |
| B4GA1 | Beta-1.4-glucuronyltransferase 1 | 11.009 | 3.87E-04 | 3.412 | 7.99E-03 |
| PIP | Prolactin-inducible protein | 11.006 | 3.87E-04 | 3.412 | 7.99E-03 |
| S12A4 | Solute carrier family 12 member 4 | 10.979 | 3.91E-04 | 3.408 | 7.99E-03 |
| TBB2A | Tubulin beta-2A chain | 10.933 | 3.98E-04 | 3.401 | 7.99E-03 |
| KLC2 | Kinesin light chain 2 | 10.929 | 3.98E-04 | 3.400 | 7.99E-03 |
| ABC3C | DNA dC->dU-editing enzyme APOBEC-3C | 10.926 | 3.98E-04 | 3.400 | 7.99E-03 |
| SPY4 | Protein sprouty homolog 4 | 10.907 | 4.01E-04 | 3.397 | 7.99E-03 |
| SREK1 | Splicing regulatory glutamine/lysine-rich protein 1 | 10.861 | 4.08E-04 | 3.389 | 7.99E-03 |

| | | | | | |
|---|---|---|---|---|---|
| EMP3 | Epithelial membrane protein 3 | 10.833 | 4.12E-04 | 3.385 | 7.99E-03 |
| SYT1; SYT2 | Extended synaptotagmin-1; Extended synaptotagmin-2 | 10.816 | 4.14E-04 | 3.383 | 7.99E-03 |
| ALDOC | Fructose-bisphosphate aldolase C | 10.816 | 4.15E-04 | 3.382 | 7.99E-03 |
| CC124 | Coiled-coil domain-containing protein 124 | 10.711 | 4.31E-04 | 3.366 | 8.23E-03 |
| TF65 | Transcription factor p65 | 10.670 | 4.37E-04 | 3.360 | 8.29E-03 |
| EXOC1 | Exocyst complex component 1 | 10.626 | 4.44E-04 | 3.353 | 8.36E-03 |
| SAR1A | GTP-binding protein SAR1a | 10.588 | 4.50E-04 | 3.347 | 8.41E-03 |
| PSMD8 | 26S proteasome non-ATPase regulatory subunit 8 | 10.510 | 4.63E-04 | 3.334 | 8.59E-03 |
| STAM1 | Signal transducing adapter molecule 1 | 10.476 | 4.69E-04 | 3.329 | 8.63E-03 |
| RS3 | 40S ribosomal protein S3 | 10.453 | 4.73E-04 | 3.325 | 8.64E-03 |
| BABA2 | BRISC and BRCA1-A complex member 2 | 10.410 | 4.81E-04 | 3.318 | 8.71E-03 |
| SC16A | Protein transport protein Sec16A | 10.377 | 4.87E-04 | 3.313 | 8.74E-03 |
| LYSC | Lysozyme C | 10.360 | 4.90E-04 | 3.310 | 8.74E-03 |
| RUXE | Small nuclear ribonucleoprotein E | 10.239 | 5.13E-04 | 3.290 | 9.08E-03 |
| ARF4 | ADP-ribosylation factor 4 | 10.203 | 5.20E-04 | 3.284 | 9.11E-03 |
| CAZA2 | F-actin-capping protein subunit alpha-2 | 10.192 | 5.22E-04 | 3.282 | 9.11E-03 |
| SPY2 | Protein sprouty homolog 2 | 10.154 | 5.30E-04 | 3.276 | 9.17E-03 |
| PURB | Transcriptional activator protein Pur-beta | 10.134 | 5.34E-04 | 3.273 | 9.18E-03 |
| EIF1; EIF1B | Eukaryotic translation initiation factor 1; Eukaryotic translation initiation factor 1b | 10.096 | 5.42E-04 | 3.266 | 9.20E-03 |
| DEK | Protein DEK | 10.090 | 5.43E-04 | 3.265 | 9.20E-03 |
| ABCF2 | ATP-binding cassette sub-family F member 2 | 10.011 | 5.60E-04 | 3.252 | 9.42E-03 |
| GGH | Gamma-glutamyl hydrolase | 9.956 | 5.72E-04 | 3.243 | 9.55E-03 |
| DC1L2 | Cytoplasmic dynein 1 light intermediate chain 2 | 9.930 | 5.78E-04 | 3.238 | 9.59E-03 |
| UCK2 | Uridine-cytidine kinase 2 | 9.912 | 5.82E-04 | 3.235 | 9.59E-03 |

* FDR: False discovery rate
