# Supplementary material for: Small Extracellular Vesicles from Hypoxic Triple-Negative Breast Cancer Cells Induce Oxygen-Dependent Cell Invasion
Source: Int J Mol Sci. 2022 Oct 21;23(20):12646. doi: 10.3390/ijms232012646 (PMC9604480; doi:10.3390/ijms232012646)
Supplement: Supplementary file 1 [file ijms-23-12646-s001.zip › Table S2.Differential proteomic profile of MDA-MB-231 cells .pdf]

Table S2. Differential proteomic profile of MDA-MB-231 cells exposed to normoxia or hypoxia, with or without SEVh treatment (p<0.05)

Protein Code	Protein Name	*f* value	*p* value	- log10(*p*)	FDR*	Tukey's HSD**
RPAB1	DNA-directed RNA polymerases I, II, and III subunit RPABC1	2900.1	1.75E-12	11.756	4.58E-09	Hyp_SEVh x Hyp_PBS<br>Hyp_SEVh x Norm_PBS<br>Norm_SEVh x Hyp_SEVh
NPL4	Nuclear protein localization protein 4 homolog	1353.2	3.68E-11	10.434	4.81E-08	Hyp_SEVh x Hyp_PBS<br>Hyp_SEVh x Norm_PBS<br>Norm_SEVh x Hyp_SEVh
KT3K	Ketosamine-3-kinase	965.53	1.41E-10	9.849	1.23E-07	Hyp_SEVh x hyp_PBS<br>Hyp_SEVh x Norm_PBS<br>Norm_SEVh x Hyp_SEVh
MRM3	rRNA methyltransferase 3, mitochondrial	746.72	3.94E-10	9.405	2.58E-07	Hyp_SEVh x Hyp_PBS<br>Hyp_SEVh x Norm_PBS<br>Norm_SEVh x Hyp_SEVh
ADDA	Alpha-adducin	681.92	5.66E-10	9.247	2.96E-07	Hyp_SEVh x Hyp_PBS<br>Hyp_SEVh x Norm_PBS<br>Norm_SEVh x Hyp_SEVh
GDE	Glycogen debranching enzyme	576.74	1.10E-09	8.958	4.80E-07	Norm_PBS x Hyp_PBS<br>Hyp_SEVh x Hyp_PBS<br>Norm_SEVh x Hyp_PBS
GMPR1	GMP reductase 1	357.98	7.33E-09	8.135	2.60E-06	Norm_PBS x Hyp_PBS<br>Hyp_SEVh x Hyp_PBS<br>Norm_SEVh x Hyp_PBS
BAG6	Large proline-rich protein BAG6	349.14	8.10E-09	8.092	2.60E-06	Hyp_SEVh x Hyp_PBS<br>Hyp_SEVh x Norm_PBS<br>Norm_SEVh x Hyp_SEVh
SETD7	Histone-lysine N-methyltransferase SETD7	335.79	9.45E-09	8.024	2.60E-06	Norm_PBS x Hyp_PBS<br>Hyp_SEVh x Hyp_PBS<br>Norm_SEVh x Hyp_PBS
GCP2	Gamma-tubulin complex component 2	331.61	9.93E-09	8.003	2.60E-06	Norm_SEVh x Hyp_PBS<br>Norm_SEVh x Norm_PBS<br>Norm_SEVh x Hyp_SEVh
CL029	Uncharacterized protein C12orf29	265.05	2.41E-08	7.617	5.74E-06	Norm_PBS x Hyp_PBS<br>Hyp_SEVh x Hyp_PBS<br>Norm_SEVh x Hyp_PBS<br>Hyp_SEVh x Norm_PBS<br>Norm_SEVh x Hyp_SEVh
KAD4	Adenylate kinase 4, mitochondrial	196.9	7.80E-08	7.108	1.70E-05	Hyp_SEVh x Hyp_PBS<br>Hyp_SEVh x Norm_PBS<br>Norm_SEVh x Hyp_SEVh
AEDO	2-aminoethanethiol dioxygenase	165.23	1.56E-07	6.808	3.07E-05	Norm_PBS x Hyp_PBS<br>Hyp_SEVh x Hyp_PBS<br>Norm_SEVh x Hyp_PBS
LYRM7	Complex III assembly factor LYRM7	162.91	1.64E-07	6.784	3.07E-05	Norm_PBS x Hyp_PBS<br>Norm_SEVh x Hyp_PBS<br>Hyp_SEVh x Norm_PBS

						Norm_SEVh x Hyp_SEVh
HSP72	Heat shock-related 70 kDa protein 2	153.85	2.06E-07	6.686	3.51E-05	Norm_PBS x Hyp_PBS<br>Hyp_SEVh x Hyp_PBS<br>Norm_SEVh x Hyp_PBS
PAPS1	Bifunctional 3'-phosphoadenosine 5'-phosphosulfate synthase 1	152.18	2.15E-07	6.668	3.51E-05	Norm_PBS x Hyp_PBS<br>Norm_SEVh x Hyp_PBS<br>Hyp_SEVh x Norm_PBS<br>Norm_SEVh x Hyp_SEVh
EPS15	Epidermal growth factor receptor substrate 15	148.61	2.36E-07	6.627	3.63E-05	Norm_PBS x Hyp_PBS<br>Hyp_SEVh x Hyp_PBS<br>Norm_SEVh x Hyp_PBS
RBX1	E3 ubiquitin-protein ligase RBX1	144.55	2.63E-07	6.580	3.82E-05	Norm_PBS x Hyp_PBS<br>Hyp_SEVh x Hyp_PBS<br>Norm_SEVh x Hyp_PBS
FBX50	F-box only protein 50	112.57	6.99E-07	6.156	9.62E-05	Norm_PBS x Hyp_PBS<br>Hyp_SEVh x Hyp_PBS<br>Norm_SEVh x Hyp_PBS
BABA1	BRISC and BRCA1-A complex member 1	104.13	9.47E-07	6.024	1.24E-04	Norm_PBS x Hyp_PBS<br>Norm_SEVh x Hyp_PBS<br>Hyp_SEVh x Norm_PBS<br>Norm_SEVh x Hyp_SEVh
TES	Testin	95.909	1.30E-06	5.885	1.61E-04	Norm_PBS x Hyp_PBS<br>Hyp_SEVh x Hyp_PBS<br>Norm_SEVh x Hyp_PBS<br>Hyp_SEVh x Norm_PBS<br>Norm_SEVh x Hyp_SEVh
GBRL2	Gamma-aminobutyric acid receptor-associated protein-like 2	94.916	1.36E-06	5.867	1.61E-04	Norm_PBS x Hyp_PBS<br>Norm_SEVh x Hyp_PBS<br>Hyp_SEVh x Norm_PBS<br>Norm_SEVh x Hyp_SEVh
KPRB	Phosphoribosyl pyrophosphate synthase-associated protein 2	88.195	1.80E-06	5.744	1.93E-04	Norm_PBS x Hyp_PBS<br>Hyp_SEVh x Norm_PBS<br>Norm_SEVh x Norm_PBS
GSTT2;GST2	Glutathione S-transferase theta-2B; Glutathione S-transferase theta-2	87.837	1.83E-06	5.737	1.93E-04	Norm_PBS x Hyp_PBS<br>Hyp_SEVh x Hyp_PBS<br>Norm_SEVh x Hyp_PBS
UCHL5	Ubiquitin carboxyl-terminal hydrolase isozyme L5	87.735	1.84E-06	5.735	1.93E-04	Hyp_SEVh x Hyp_PBS<br>Hyp_SEVh x Norm_PBS<br>Norm_SEVh x Hyp_SEVh
DDX23	Probable ATP-dependent RNA helicase DDX23	86.176	1.97E-06	5.705	1.98E-04	Hyp_SEVh x Hyp_PBS<br>Hyp_SEVh x Norm_PBS<br>Norm_SEVh x Hyp_SEVh
COA6	Cytochrome c oxidase assembly factor 6 homolog	82.576	2.33E-06	5.633	2.25E-04	Hyp_SEVh x Hyp_PBS<br>Hyp_SEVh x Norm_PBS<br>Norm_SEVh x Hyp_SEVh

BID	BH3-interacting domain death agonist	79.736	2.66E-06	5.575	2.46E-04	Norm_PBS x Hyp_PBS<br>Norm_SEVh x Hyp_PBS<br>Hyp_SEVh x Norm_PBS<br>Norm_SEVh x Hyp_SEVh
P4HA2	Prolyl 4-hydroxylase subunit alpha-2	79.284	2.72E-06	5.565	2.46E-04	Norm_PBS x Hyp_PBS<br>Norm_SEVh x Hyp_PBS<br>Hyp_SEVh x Norm_PBS<br>Norm_SEVh x Hyp_SEVh
CSRP2	Cysteine and glycine-rich protein 2	74.86	3.40E-06	5.469	2.92E-04	Norm_PBS x Hyp_PBS<br>Norm_SEVh x Hyp_PBS<br>Hyp_SEVh x Norm_PBS<br>Norm_SEVh x Hyp_SEVh
NEUA	N-acylneuraminate cytidylyltransferase	74.487	3.46E-06	5.461	2.92E-04	Norm_PBS x Hyp_PBS<br>Hyp_SEVh x Hyp_PBS<br>Norm_SEVh x Hyp_PBS<br>Hyp_SEVh x Norm_PBS<br>Norm_SEVh x Hyp_SEVh
DBR1	Lariat debranching enzyme	73.861	3.58E-06	5.446	2.92E-04	Norm_PBS x Hyp_PBS<br>Hyp_SEVh x Norm_PBS<br>Norm_SEVh x Norm_PBS
PORIM	Porimin	63.112	6.54E-06	5.185	5.18E-04	Norm_PBS x Hyp_PBS<br>Norm_SEVh x Hyp_PBS<br>Hyp_SEVh x Norm_PBS<br>Norm_SEVh x Hyp_SEVh
CYTC	Cystatin-C	62.636	6.73E-06	5.172	5.18E-04	Norm_PBS x Hyp_PBS<br>Hyp_SEVh x Norm_PBS<br>Norm_SEVh x Norm_PBS
CKAP2	Cytoskeleton-associated protein 2	61.221	7.34E-06	5.134	5.49E-04	Norm_PBS x Hyp_PBS<br>Norm_SEVh x Hyp_PBS<br>Hyp_SEVh x Norm_PBS<br>Norm_SEVh x Hyp_SEVh
SNX12	Sorting nexin-12	58.753	8.59E-06	5.066	6.19E-04	Norm_PBS x Hyp_PBS<br>Hyp_SEVh x Norm_PBS<br>Norm_SEVh x Norm_PBS
CGL	Cystathionine gamma-lyase	58.069	8.98E-06	5.047	6.19E-04	Norm_PBS x Hyp_PBS<br>Hyp_SEVh x Hyp_PBS<br>Norm_SEVh x Hyp_PBS
ACYP1	Acylphosphatase-1	58.043	9.00E-06	5.046	6.19E-04	Norm_PBS x Hyp_PBS<br>Norm_SEVh x Hyp_PBS<br>Hyp_SEVh x Norm_PBS<br>Norm_SEVh x Hyp_SEVh
CD47	Leukocyte surface antigen CD47	57.592	9.27E-06	5.033	6.22E-04	Hyp_SEVh x Hyp_PBS<br>Hyp_SEVh x Norm_PBS<br>Norm_SEVh x Hyp_SEVh
ATG3	Ubiquitin-like-conjugating enzyme ATG3	57.135	9.55E-06	5.020	6.25E-04	Norm_PBS x Hyp_PBS<br>Norm_SEVh x Hyp_PBS<br>Hyp_SEVh x Norm_PBS<br>Norm_SEVh x Hyp_SEVh

KITH	Thymidine kinase, cytosolic	55.105	1.10E-05	4.960	6.99E-04	Norm_PBS x Hyp_PBS<br>Norm_SEVh x Hyp_PBS<br>Hyp_SEVh x Norm_PBS<br>Norm_SEVh x Hyp_SEVh
SRRT	Serrate RNA effector molecule homolog	54.119	1.17E-05	4.930	7.31E-04	Norm_PBS x Hyp_PBS<br>Hyp_SEVh x Norm_PBS<br>Norm_SEVh x Norm_PBS
PCY2	Ethanolamine-phosphate cytidylyltransferase	52.442	1.32E-05	4.879	7.95E-04	Norm_PBS x Hyp_PBS<br>Hyp_SEVh x Hyp_PBS<br>Norm_SEVh x Hyp_PBS
FKBP9	Peptidyl-prolyl cis-trans isomerase FKBP9	52.28	1.34E-05	4.873	7.95E-04	Norm_PBS x Hyp_PBS<br>Norm_SEVh x Hyp_PBS<br>Hyp_SEVh x Norm_PBS<br>Norm_SEVh x Hyp_SEVh
PPCEL	Prolyl endopeptidase-like	51.713	1.39E-05	4.856	8.11E-04	Norm_PBS x Hyp_PBS<br>Hyp_SEVh x Hyp_PBS<br>Norm_SEVh x Hyp_PBS
PAPS2	Bifunctional 3'-phosphoadenosine 5'-phosphosulfate synthase 2	47.31	1.95E-05	4.710	1.11E-03	Norm_PBS x Hyp_PBS<br>Norm_SEVh x Hyp_PBS<br>Hyp_SEVh x Norm_PBS<br>Norm_SEVh x Hyp_SEVh
PP1R7	Protein phosphatase 1 regulatory subunit 7	46.568	2.07E-05	4.684	1.15E-03	Norm_PBS x Hyp_PBS<br>Norm_SEVh x Hyp_PBS<br>Hyp_SEVh x Norm_PBS<br>Norm_SEVh x Hyp_SEVh
GAL3B;GAL3A	Glutamine amidotransferase-like class 1 domain-containing protein 3, mitochondrial; Putative glutamine amidotransferase-like class 1 domain-containing protein 3B, mitochondrial	40.451	3.51E-05	4.455	1.91E-03	Norm_PBS x Hyp_PBS<br>Norm_SEVh x Hyp_PBS<br>Hyp_SEVh x Norm_PBS<br>Norm_SEVh x Hyp_SEVh
HBE	Hemoglobin subunit epsilon	39.618	3.79E-05	4.421	2.02E-03	Norm_PBS x Hyp_PBS<br>Hyp_SEVh x Hyp_PBS<br>Norm_SEVh x Hyp_PBS
CHSP1	Calcium-regulated heat-stable protein 1	38.468	4.23E-05	4.373	2.21E-03	Norm_PBS x Hyp_PBS<br>Hyp_SEVh x Hyp_PBS<br>Norm_SEVh x Hyp_PBS
YLPM1	YLP motif-containing protein 1	33.801	6.83E-05	4.166	3.50E-03	Hyp_SEVh x Hyp_PBS<br>Hyp_SEVh x Norm_PBS<br>Norm_SEVh x Hyp_SEVh
GALK1	Galactokinase	32.469	7.91E-05	4.102	3.98E-03	Norm_PBS x Hyp_PBS<br>Norm_SEVh x Hyp_PBS<br>Hyp_SEVh x Norm_PBS<br>Norm_SEVh x Hyp_SEVh
PICAL	Phosphatidylinositol-binding clathrin assembly protein	29.018	1.19E-04	3.924	5.88E-03	Norm_PBS x Hyp_PBS<br>Norm_SEVh x Hyp_PBS<br>Hyp_SEVh x Norm_PBS<br>Norm_SEVh x Hyp_SEVh

STABP	STAM-binding protein	28.388	1.29E-04	3.889	6.25E-03	Norm_PBS x Hyp_PBS<br>Norm_SEVh x Hyp_PBS<br>Hyp_SEVh x Norm_PBS<br>Norm_SEVh x Hyp_SEVh
GORS2	Golgi reassembly-stacking protein 2	26.761	1.60E-04	3.796	7.60E-03	Norm_PBS x Hyp_PBS<br>Norm_SEVh x Hyp_PBS<br>Hyp_SEVh x Norm_PBS<br>Norm_SEVh x Hyp_SEVh
GTR3;GTR14	Solute carrier family 2, facilitated glucose transporter member 3; Solute carrier family 2, facilitated glucose transporter member 14	25.888	1.80E-04	3.745	8.41E-03	Norm_PBS x Hyp_PBS<br>Hyp_SEVh x Hyp_PBS<br>Norm_SEVh x Hyp_PBS
RPB9	DNA-directed RNA polymerase II subunit RPB9	25.742	1.84E-04	3.736	8.43E-03	Norm_PBS x Hyp_PBS<br>Hyp_SEVh x Hyp_PBS<br>Norm_SEVh x Hyp_PBS
KNTC1	Kinetochore-associated protein 1	25.62	1.87E-04	3.728	8.43E-03	Norm_PBS x Hyp_PBS<br>Hyp_SEVh x Hyp_PBS<br>Norm_SEVh x Hyp_PBS
ANLN	Anillin	25.319	1.95E-04	3.710	8.64E-03	Norm_PBS x Hyp_PBS<br>Norm_SEVh x Hyp_PBS<br>Hyp_SEVh x Norm_PBS<br>Norm_SEVh x Hyp_SEVh
TAGL	Transgelin	24.388	2.23E-04	3.652	9.72E-03	Hyp_SEVh x Hyp_PBS<br>Hyp_SEVh x Norm_PBS<br>Norm_SEVh x Hyp_SEVh
ZN638	Zinc finger protein 638	24.009	2.36E-04	3.628	9.99E-03	Norm_SEVh x Hyp_PBS<br>Norm_SEVh x Norm_PBS<br>Norm_SEVh x Hyp_SEVh
CHCH2;CHCH9	Coiled-coil-helix-coiled-coil-helix domain-containing protein 2; Putative coiled-coil-helix-coiled-coil-helix domain-containing protein CHCHD2P9, mitochondrial	23.975	2.37E-04	3.625	9.99E-03	Norm_PBS x Hyp_PBS<br>Norm_SEVh x Hyp_PBS<br>Hyp_SEVh x Norm_PBS<br>Norm_SEVh x Hyp_SEVh
ERO1A	ERO1-like protein alpha	23.765	2.44E-04	3.612	1.01E-02	Norm_PBS x Hyp_PBS<br>Norm_SEVh x Hyp_PBS<br>Hyp_SEVh x Norm_PBS<br>Norm_SEVh x Hyp_SEVh
BAF	Barrier-to-autointegration factor	23.567	2.52E-04	3.599	1.03E-02	Norm_SEVh x Hyp_PBS<br>Norm_SEVh x Norm_PBS<br>Norm_SEVh x Hyp_SEVh
OSBP1	Oxysterol-binding protein 1	22.995	2.75E-04	3.561	1.11E-02	Norm_PBS x Hyp_PBS<br>Norm_SEVh x Hyp_PBS<br>Hyp_SEVh x Norm_PBS<br>Norm_SEVh x Hyp_SEVh
CNPY3	Protein canopy homolog 3	22.488	2.97E-04	3.527	1.17E-02	Norm_PBS x Hyp_PBS<br>Norm_SEVh x Hyp_PBS<br>Hyp_SEVh x Norm_PBS

						Norm_SEVh x Hyp_SEVh
CARM1	Histone-arginine methyltransferase CARM1	22.373	3.03E-04	3.519	1.17E-02	Norm_PBS x Hyp_PBS<br>Hyp_SEVh x Norm_PBS<br>Norm_SEVh x Norm_PBS
ZA2G	Zinc-alpha-2-glycoprotein	22.317	3.05E-04	3.515	1.17E-02	Norm_PBS x Hyp_PBS<br>Norm_SEVh x Hyp_PBS<br>Hyp_SEVh x Norm_PBS<br>Norm_SEVh x Hyp_SEVh
HAT1	Histone acetyltransferase type B catalytic subunit	22.065	3.18E-04	3.498	1.20E-02	Norm_PBS x Hyp_PBS<br>Norm_SEVh x Hyp_PBS<br>Hyp_SEVh x Norm_PBS<br>Norm_SEVh x Hyp_SEVh
IDI1	Isopentenyl-diphosphate Delta-isomerase 1	21.991	3.22E-04	3.493	1.20E-02	Norm_PBS x Hyp_PBS<br>Norm_SEVh x Hyp_PBS<br>Hyp_SEVh x Norm_PBS<br>Norm_SEVh x Hyp_SEVh
UBA6	Ubiquitin-like modifier-activating enzyme 6	21.789	3.32E-04	3.478	1.22E-02	Norm_PBS x Hyp_PBS<br>Hyp_SEVh x Norm_PBS<br>Norm_SEVh x Norm_PBS
DNM1L	Dynamin-1-like protein	21.661	3.39E-04	3.469	1.23E-02	Norm_PBS x Hyp_PBS<br>Hyp_SEVh x Norm_PBS<br>Norm_SEVh x Norm_PBS
TF65	Transcription factor p65	21.325	3.58E-04	3.446	1.28E-02	Norm_PBS x Hyp_PBS<br>Norm_SEVh x Hyp_PBS<br>Hyp_SEVh x Norm_PBS<br>Norm_SEVh x Hyp_SEVh
TOPK	Lymphokine-activated killer T-cell-originated protein kinase	21.272	3.62E-04	3.442	1.28E-02	Norm_PBS x Hyp_PBS<br>Norm_SEVh x Hyp_PBS<br>Hyp_SEVh x Norm_PBS<br>Norm_SEVh x Hyp_SEVh
HPF1	Histone PARylation factor 1	20.788	3.92E-04	3.407	1.35E-02	Hyp_SEVh x Hyp_PBS<br>Hyp_SEVh x Norm_PBS<br>Norm_SEVh x Hyp_SEVh
DLDH	Dihydrolipoyl dehydrogenase, mitochondrial	20.767	3.93E-04	3.405	1.35E-02	Norm_PBS x Hyp_PBS<br>Norm_SEVh x Hyp_PBS<br>Hyp_SEVh x Norm_PBS<br>Norm_SEVh x Hyp_SEVh
IPP2;IPP2B	Protein phosphatase inhibitor 2; Protein phosphatase inhibitor 2 family member B	20.638	4.02E-04	3.396	1.37E-02	Norm_PBS x Hyp_PBS<br>Hyp_SEVh x Norm_PBS<br>Norm_SEVh x Norm_PBS
SAP18	Histone deacetylase complex subunit SAP18	19.968	4.51E-04	3.346	1.51E-02	Hyp_SEVh x Hyp_PBS<br>Hyp_SEVh x Norm_PBS<br>Norm_SEVh x Hyp_SEVh
RL26	60S ribosomal protein L26	19.904	4.56E-04	3.341	1.51E-02	Norm_PBS x Hyp_PBS<br>Norm_SEVh x Hyp_PBS<br>Hyp_SEVh x Norm_PBS

						Norm_SEVh x Hyp_SEVh
IDHP	Isocitrate dehydrogenase [NADP], mitochondrial	19.478	4.92E-04	3.308	1.61E-02	Norm_PBS x Hyp_PBS<br>Hyp_SEVh x Norm_PBS<br>Norm_SEVh x Norm_PBS
H12	Histone H1.2	18.579	5.79E-04	3.237	1.87E-02	Norm_PBS x Hyp_PBS<br>Norm_SEVh x Hyp_PBS<br>Hyp_SEVh x Norm_PBS<br>Norm_SEVh x Hyp_SEVh
MD2L1	Mitotic spindle assembly checkpoint protein MAD2A	17.398	7.26E-04	3.139	2.31E-02	Hyp_SEVh x Hyp_PBS<br>Hyp_SEVh x Norm_PBS<br>Norm_SEVh x Hyp_SEVh
TBB2A	Tubulin beta-2A chain	16.957	7.92E-04	3.101	2.46E-02	Norm_PBS x Hyp_PBS<br>Norm_SEVh x Hyp_PBS<br>Hyp_SEVh x Norm_PBS<br>Norm_SEVh x Hyp_SEVh
GSH1	Glutamate--cysteine ligase catalytic subunit	16.924	7.97E-04	3.098	2.46E-02	Norm_PBS x Hyp_PBS<br>Norm_SEVh x Hyp_PBS<br>Hyp_SEVh x Norm_PBS<br>Norm_SEVh x Hyp_SEVh
ACO13	Acyl-coenzyme A thioesterase 13	16.919	7.98E-04	3.098	2.46E-02	Norm_PBS x Hyp_PBS<br>Hyp_SEVh x Norm_PBS<br>Norm_SEVh x Norm_PBS
AIP	AH receptor-interacting protein	16.711	8.33E-04	3.080	2.53E-02	Norm_PBS x Hyp_PBS<br>Hyp_SEVh x Norm_PBS<br>Norm_SEVh x Norm_PBS
PQBP1	Polyglutamine-binding protein 1	16.431	8.82E-04	3.055	2.65E-02	Hyp_SEVh x Hyp_PBS<br>Hyp_SEVh x Norm_PBS<br>Norm_SEVh x Hyp_SEVh
NDRG1	Protein NDRG1	16.27	9.12E-04	3.040	2.71E-02	Norm_PBS x Hyp_PBS<br>Norm_SEVh x Hyp_PBS<br>Hyp_SEVh x Norm_PBS<br>Norm_SEVh x Hyp_SEVh
DSC1	Desmocollin-1	16.051	9.54E-04	3.020	2.80E-02	Norm_PBS x Hyp_PBS<br>Norm_SEVh x Hyp_PBS<br>Hyp_SEVh x Norm_PBS<br>Norm_SEVh x Hyp_SEVh
F107B	Protein FAM107B	15.403	1.10E-03	2.960	3.15E-02	Norm_PBS x Hyp_PBS<br>Norm_SEVh x Hyp_PBS<br>Hyp_SEVh x Norm_PBS<br>Norm_SEVh x Hyp_SEVh
MUCL1	Mucin-like protein 1	15.397	1.10E-03	2.960	3.15E-02	Norm_PBS x Hyp_PBS<br>Hyp_SEVh x Hyp_PBS<br>Norm_SEVh x Hyp_PBS
PDIA1	Protein disulfide-isomerase	15.129	1.16E-03	2.934	3.31E-02	Norm_PBS x Hyp_PBS<br>Norm_SEVh x Hyp_PBS<br>Hyp_SEVh x Norm_PBS

SRP68	Signal recognition particle subunit SRP68	14.926	1.22E-03	2.914	3.42E-02	Norm_PBS x Hyp_PBS<br>Norm_SEVh x Hyp_PBS<br>Hyp_SEVh x Norm_PBS<br>Norm_SEVh x Hyp_SEVh
COASY	Bifunctional coenzyme A synthase	14.79	1.26E-03	2.901	3.49E-02	Hyp_SEVh x Hyp_PBS<br>Hyp_SEVh x Norm_PBS<br>Norm_SEVh x Hyp_SEVh
BPNT1	3'(2'),5'-bisphosphate nucleotidase 1	14.572	1.32E-03	2.880	3.63E-02	Norm_PBS x Hyp_PBS<br>Hyp_SEVh x Hyp_PBS<br>Norm_SEVh x Norm_PBS
RL1D1	Ribosomal L1 domain-containing protein 1	14.084	1.48E-03	2.831	4.02E-02	Norm_PBS x Hyp_PBS<br>Norm_SEVh x Hyp_PBS<br>Hyp_SEVh x Norm_PBS<br>Norm_SEVh x Hyp_SEVh
UBR7	Putative E3 ubiquitin-protein ligase UBR7	13.988	1.51E-03	2.821	4.07E-02	Norm_PBS x Hyp_PBS<br>Hyp_SEVh x Norm_PBS<br>Norm_SEVh x Norm_PBS
ASNS	Asparagine synthetase [glutamine-hydrolyzing]	13.848	1.56E-03	2.807	4.17E-02	Norm_PBS x Hyp_PBS<br>Norm_SEVh x Hyp_PBS<br>Hyp_SEVh x Norm_PBS<br>Norm_SEVh x Hyp_SEVh
NQO2	Ribosyldihydronicotinamide dehydrogenase [quinone]	13.381	1.75E-03	2.758	4.62E-02	Norm_PBS x Hyp_PBS<br>Hyp_SEVh x Norm_PBS
UBP7	Ubiquitin carboxyl-terminal hydrolase 7	13.046	1.90E-03	2.722	4.96E-02	Norm_PBS x Hyp_PBS<br>Norm_SEVh x Hyp_PBS<br>Norm_SEVh x Hyp_SEVh

* FDR: False discovery rate; ** Tukey’s HSD: Tukey’s honest significant difference
