# Supplementary figures and images for: Small Extracellular Vesicles from Hypoxic Triple-Negative Breast Cancer Cells Induce Oxygen-Dependent Cell Invasion
Source: Int J Mol Sci. 2022 Oct 21;23(20):12646. doi: 10.3390/ijms232012646 (PMC9604480; doi:10.3390/ijms232012646)

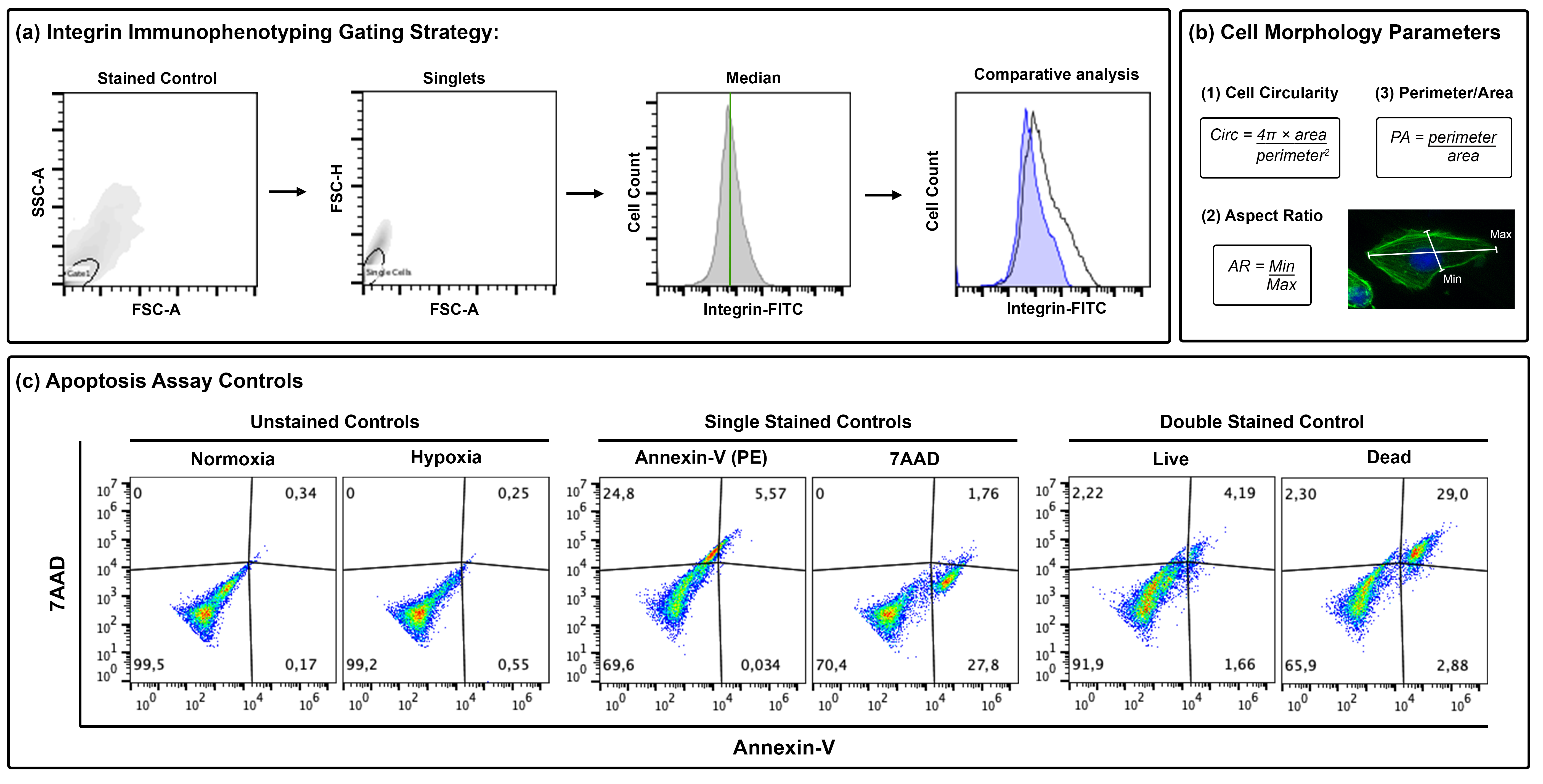

Supplement: Supplementary file 1 [file ijms-23-12646-s001.zip › Figure S1. Additional Methods.tif]

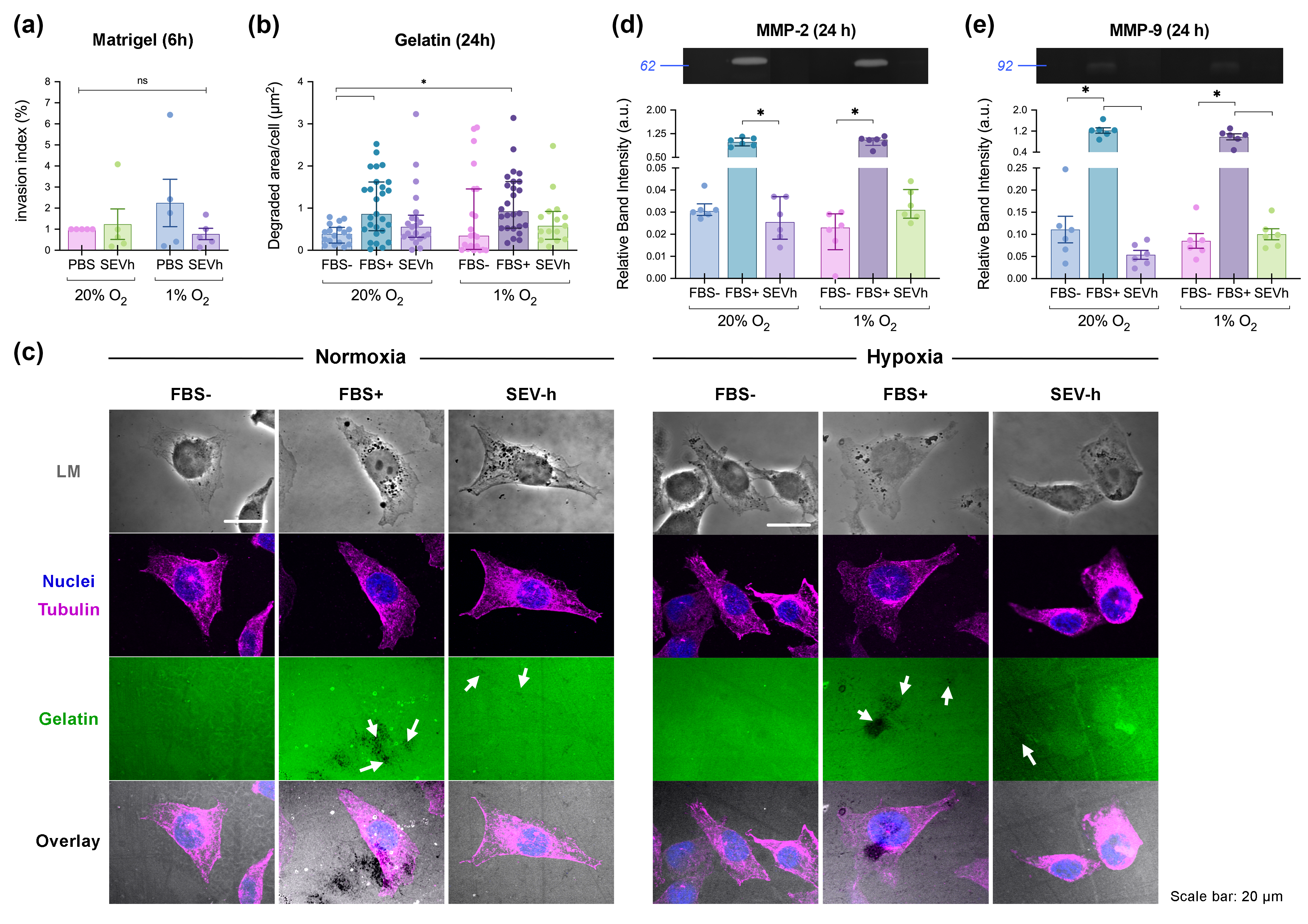

Supplement: Supplementary file 1 [file ijms-23-12646-s001.zip › Figure S2. Invasion 24h.tif]

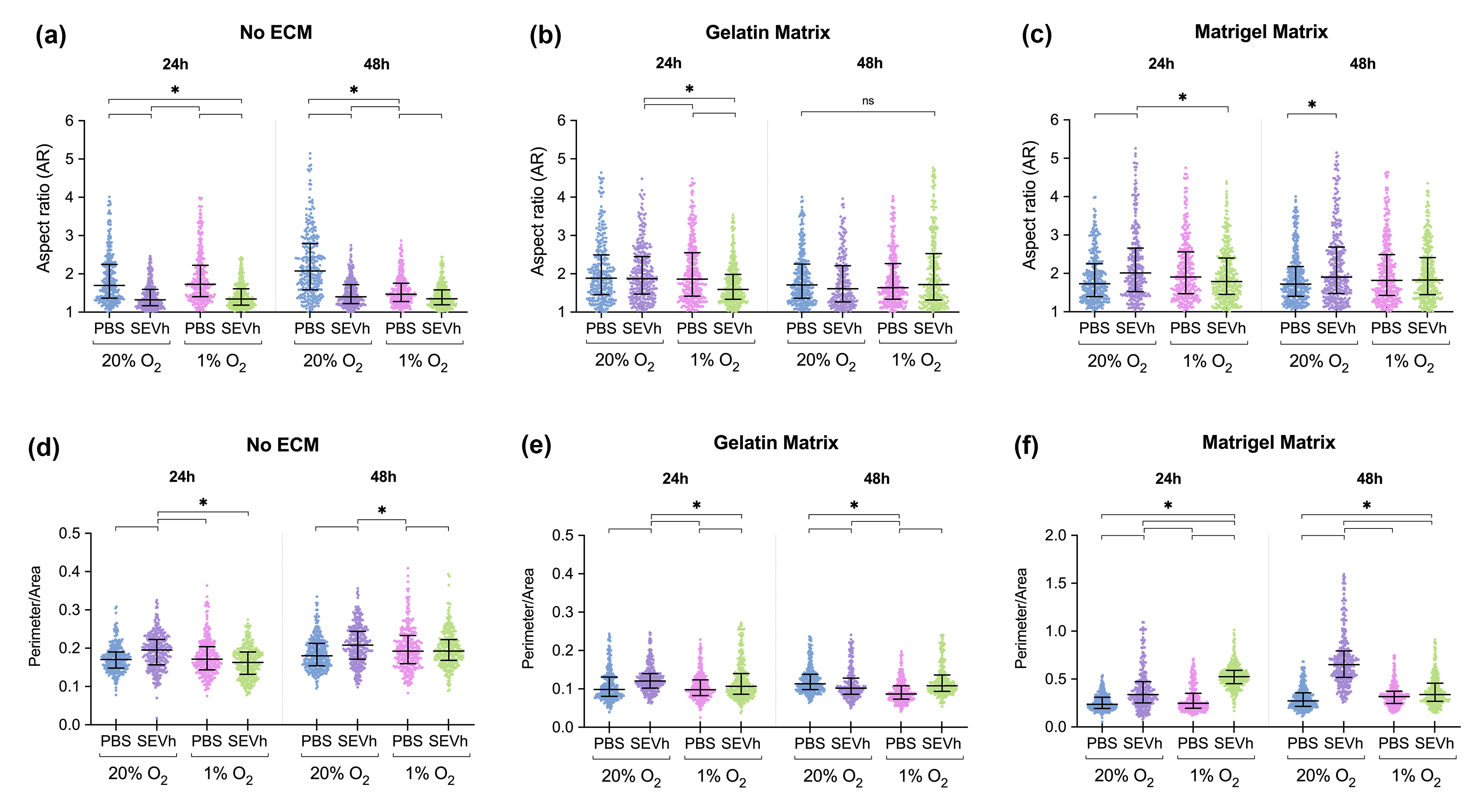

Supplement: Supplementary file 1 [file ijms-23-12646-s001.zip › Figure S3. Morphology.tif]
